# Duration of Pulpless Teeth

**Published:** 1896-07

**Authors:** 

**Affiliations:** Cincinnati, Ohio.


ARTICLE V.

### DURATION OF PULPLESS TEETH.

DR. TAFT, CINCINNATI, OHIO.

The tooth derives its nutritive supply from the pulp and it goes on from its primary condition to its perfection (speaking of the permanent teeth); but what about it after this period? Is it of any value after the perfection of the tooth? Were it not of value in the economy, Nature would have made provision for its removal. Does the tooth need any nutrition after twenty-five years of age, after it has been entirely calcified? Can any one decide in any given case when the period of perfect calcification is reached? It is reached much earlier in some cases than in others. It is a fact patent to every close observer that the teeth in many instances do not seem to be complete in calcification at thirty or thirty-five years of age. How do we know? Up to this time they have remained in a comparatively deficient condition in this respect. They become more and more dense after this time. Does that increased solidification take place after the pulp is destroyed? Two general classes, organic and inorganic, vital and non-vital, the vital just as important an element as the non-vital. The vital must have supply and nutrition. When its life is taken away, deterioration at once begins, not in the broad sense manifesting disintegration at once, though that comes afterward. The organic portion of the tooth is not nourished as when the pulp is living. Both the dentine and enamel receive their supply from the pulp of the tooth, and when this organ is destroyed this process of nutrition is also destroyed. It will be found that after a pulp is taken away, disintegration and breaking down of the tooth takes place without the ordinary process of decay, simply by a deterioration of the tooth-structure. That, of course, appears on the organic material, and not the inorganic. Often we find a devitalized tooth with a portion of it broken off. The enamel far more readily breaks down, and disintegration occurs wherever a thin edge is left, or where there is an exposed edge or border, because of its weakened condition. That is true also of the dentin, because that has within it a much larger proportion of organic matter. I remember the case of an excellent tooth in a mouth in which all the teeth were excellent, except that the first permanent molar by decay had lost its pulp. There was a large cavity running through from front to back. On that tooth Dr. Atkinson many years ago performed an operation, filling up the pulp-chamber and building up the tooth. The statement was made that the tooth would last the lifetime of the patient, but it did not. It was a firm, solid tooth, as was its neighbors. That tooth lasted about eighteen years, and did good service, except about twelve or thirteen years after it had been filled the edges of the enamel began to break away. Examination revealed the fact that there was no ordinary decay. It was very slightly calcified, but there had been such deterioration of the organic portion of the tooth that it was not able to withstand the force that was put on it in mastication and gave way. Within a year the other side broke off in the same manner, showing clearly that there was a deterioration of the structure or character of the tooth that was fatal to it.—*International.*

